# Advances in the Regulation of Periostin for Osteoarthritic Cartilage Repair Applications

**DOI:** 10.3390/biom14111469

**Published:** 2024-11-18

**Authors:** Sunny Y. Shih, Michael P. Grant, Laura M. Epure, Muskan Alad, Sophie Lerouge, Olga L. Huk, Stephane G. Bergeron, David J. Zukor, Géraldine Merle, Hee-Jeong Im, John Antoniou, Fackson Mwale

**Affiliations:** 1 Department of Surgical and Interventional Sciences, Faculty of Medicine and Health Sciences, McGill University, Montreal, QC H3G 2M1, Canadageraldine.merle@mcgill.ca (G.M.);; 2 Orthopaedic Research Laboratory, Lady Davis Institute for Medical Research, Montreal, QC H3T 1E2, Canada; 3 Department of Orthopaedics, SMBD-Jewish General Hospital, McGill University, Montreal, QC H3T 1E2, Canada; 4 Department of Mechanical Engineering, École de Technologie Supérieure (ETS), Montreal, QC H3C 1K3, Canada; 5 Laboratory of Endovascular Biomaterials (LBeV), Centre de Recherche du CHUM (CRCHUM), Montreal, QC H2X 0A9, Canada; 6 Chemical Engineering Department, Polytechnique Montréal, Montreal, QC H3C 3A7, Canada; 7 Department of Bioengineering, University of Illinois at Chicago, Chicago, IL 60607, USA; 8 Jesse Brown Veterans Affairs Medical Center (JBVAMC), Chicago, IL 60612, USA

**Keywords:** periostin, cartilage, chondrocyte, Link N, osteoarthritis

## Abstract

Emerging evidence indicates periostin (POSTN) is upregulated in patients with OA, and studies have shown that it can induce the activation of inflammatory cytokines and catabolic enzymes, making it a potential therapeutic target. Link N (LN) is a peptide fragment derived from the link protein and has been demonstrated as an anabolic-like factor and anti-catabolic and anti-inflammatory factors both in vitro and in vivo. This study aims to determine if LN can regulate POSTN expression and function in OA cartilage. Articular cartilage was recovered from donors undergoing total knee replacements to isolate chondrocytes and prepare osteochondral explants. Cells and explants were treated with POSTN and LN (1 and 100 μg) and measured for changes in POSTN expression and various matrix proteins, catabolic and proinflammatory factors, and signaling. To determine the effects of POSTN expression in vivo, a rabbit OA model was used. Immunoprecipitation and in silico modeling were used to determine peptide/POSTN interactions. Western blotting, PCR, and immunohistochemistry demonstrated that LN decreased POSTN expression both in vitro and in vivo. LN was also able to directly inhibit POSTN signaling in OA chondrocytes. In silico docking suggested the direct interaction of LN with POSTN at residues responsible for its oligomerization. Immunoprecipitation experiments confirmed the direct interaction of LN with POSTN and the destabilization of its oligomerization. This study demonstrates the ability of a peptide, LN, to suppress the overexpression and function of POSTN in OA cartilage.

## 1. Introduction

Osteoarthritis (OA) is one of the leading causes of disability in the adult population and is characterized by disruption in the structural and functional properties of articular joints [1]. Although the etiology of the disease is unclear, it is widely accepted that these degenerative changes arise from the imbalance of synthetic and degradative pathways that control the metabolism of the cartilage extracellular matrix (ECM) [2]. As cartilage has a limited capacity for self-regeneration, OA remains an incurable degenerative joint disorder characterized by two fundamental pathological changes: the inflammation and destruction of the articular cartilage [2]. Although several factors have been implicated in the development of OA, periostin (POSTN) has recently been described to play a contributing role [3,4,5].

POSTN is a 93.3 kDa secreted matricellular protein believed to function as a dimer or higher-order oligomer [6]. It is expressed in bone and collagen-rich fibrous connective tissues and is a member of the fasciclin [7] family of proteins, also known as osteoblast-specific factor 2 (OSF2). POSTN has a multi-domain structure, allowing for it to interact with cell surface receptors, proteases, and other molecules that regulate cellular adhesion, collagen fibrillogenesis, and the promotion of ECM crosslinking [8]. This protein is widely expressed in various tissues and cells and has been implicated in several physiological systems, including musculoskeletal, cardiovascular, and respiratory.

The increased expression of POSTN following an injury appears to be detrimental to articular cartilage, as it stimulates a catabolic cascade in chondrocytes and may contribute to the development of OA [3]. POSTN expression is induced by transforming growth factor (TGF)-b and functions as a ligand for alpha-V/beta-3 and alpha-V/beta-5 integrins in osteoblast, fibroblast, and epithelial cells [9,10,11]. These interactions can activate mitogen-activated protein kinase (MAPK), leading to increased NF-kB signaling, dysregulation of Wnt via crosstalk with other surface receptors [12,13] and hedgehog and TGF-β signaling, thought to be involved in the progression of OA. Interestingly, POSTN- the induced matrix metalloproteinase (MMP)-13 expression, a hallmark of OA, was inhibited by CCT031374 hydrobromide, an inhibitor of the canonical Wnt/beta-catenin-signaling pathway [14]. These specific findings suggest that targeting POSTN has therapeutic value in the treatment of OA. Despite the importance of POSTN in the development of OA, to date, there are no known targeted peptide inhibitors.

Although POSTN is primarily known for its role in the extracellular matrix, it also exists within the cytosol and has a nuclear localization signal, indicating potential intracellular functions. This study focuses on the extracellular actions of POSTN in OA, where it interacts with matrix components and receptors, driving cartilage degradation. The targeted inhibition of LN on extracellular POSTN offers distinct advantages over intracellular suppression strategies, such as siRNA or antisense oligonucleotides.

Link N (DHLSDNYTLDHDRAIH, LN) is a 16-residue peptide fragment derived from the proteolytic processing of Link protein, an extracellular matrix glycoprotein that stabilizes proteoglycan aggregates in cartilage and the intervertebral disk by binding to both hyaluronic acid and aggrecan [15,16].

We, and others, have demonstrated that LN can behave as an anabolic-like factor by stimulating matrix protein synthesis in cartilage and the intervertebral disk [15,17,18,19,20,21,22,23]. LN has also been shown to inhibit the catabolic effects of IL-1β on cartilage and intervertebral disk tissues [24,25]. Additionally, LN was found to modulate inflammation and stimulate the repair of arthritis-mediated temporomandibular joint disruption in vivo [26]. Moreover, the delivery of LN-encoded mRNA into primary human chondrocytes and mesenchymal stromal cells resulted in enhanced expressions of aggrecan, Sox 9, and type II collagen [26]. In a similar report, LN was found to stimulate the chondrogenic differentiation of cartilage stem cell progenitor cells [27]. In an in vivo OA models, the intraarticular injection of LN significantly reduced the disease burden and pain [24,28]. 

As pointed out, POSTN is a matricellular protein that plays a critical role in tissue remodeling and repair processes under normal physiological conditions [29]. However, its overexpression in OA cartilage contributes significantly to the pathological progression of the disease. Targeting POSTN for therapeutic intervention, therefore, focuses on its upregulated state in OA rather than its physiological expression in healthy tissues. This study aims to determine if LN can regulate POSTN expression and function in OA cartilage, thereby potentially mitigating catabolic signaling and inflammatory responses associated with OA progression.

## 2. Materials and Methods

### 2.1. Reagents

Pierce Avidin agarose beads were purchased from ThermoFisher (Cat# 20219, Waltham, MA, USA). Recombinant human POSTN protein was purchased from Abcam (Cat# ab203522, Cambridge, UK).

### 2.2. Peptide Synthesis

Link N (DHLSDNYTLDHDRAIH) and short Link N (sLN; DHLSDNYT) peptides were synthesized by CanPeptide (Pointe Claire, QC, Canada). Scrambled Link N (SC; DLNRAHLHIDYHTDSD) was designed using a bioinformatics tool from Institut Pasteur, Paris, France (http://mobyle.pasteur.fr (accessed on 13 May 2021)). Biotinylated LN and SC were synthesized and prepared by BioMatik Corporation (Kitchener, ON, Canada).

### 2.3. Antibodies

Anti-periostin antibody (ThermoFisher Scientific), anti-phospho-β-catenin antibody (Abcam), and POSTN mouse monoclonal antibody (ThermoFisher Scientific, Waltham, MA, USA, Cat# 66491-1Ig) were used.

### 2.4. Rabbit ACL Transection of an OA Model

An anterior cruciate ligament transection of an (ACLT) OA model was conducted with the approval of the Institutional Animal Care and Use Committee in accordance with the Canadian Council on Animal Care. Twenty-two skeletally mature (8-month-old) female New Zealand white rabbits (Charles River, St. Constant, QC, Canada) with a mean weight of 3.9 kg (range: 3.4–5.1 kg) were used for this study. The rabbits were randomly divided into 3 groups: 8 rabbits for ACLT followed by sLN treatment, 8 rabbits for ACLT followed by saline treatment, and 6 Sham-operated controls without ACLT or any further treatments. Unilateral ACLT surgeries were performed on 16 rabbits on Day 0. Treatment was administrated at 3, 6, and 9 weeks post ACLT by intraarticular injections into the operated knee. The sLN group (*n* = 8 rabbits) received 100 µg of LN in 1 mL of saline, while the saline group (*n* = 8) received 1 mL of saline (vehicle). All the animals were sacrificed 12 weeks post ACLT surgery. All the surgeries and intraarticular injections were performed by a board-certified veterinary surgeon, as previously described [28].

Following euthanasia, the treated femorotibial joints were harvested and fixed in 10% neutral-buffered formalin. Subsequently, sagittal sections centered on the most severe lesion were cut from each treated joint using an ISOMET saw (Buehler, Lake Bluff, IL, USA) equipped with a 15 HC diamond blade (10.2 mm × 0.3 mm). Tissue blocks were decalcified for 2 weeks with a 14% ethylenediaminetetraacetic acid solution (Fisher Scientific, Nepean, ON, Canada), dehydrated through graded alcohols, and cleared with toluol prior to being embedded in paraffin wax. Multiple 5 µm sections were cut from the tissue blocks and used for the immunohistochemistry assessment.

### 2.5. Human OA Cartilage Explant Preparation and Treatments

Osteochondral explants were obtained from joints of OA donors undergoing total replacement surgeries. Normal cartilage explants were purchased from Advanced Tissue Services (Tucson, AZ, USA). Explants containing both cartilage and bone were cut using a circular saw and bone cutters to approximately 1 cm^2^ in size and then washed thrice in phosphate-buffered saline (PBS) containing penicillin–streptomycin (Wisent Bioproducts, Montreal, QC, Canada), followed by a 30 min incubation with 0.125% trypsin (Wisent Bioproducts, Montreal, QC, Canada) in low-glucose Dulbecco’s Modified Eagle Medium (DMEM, Wisent Bioproducts, Montreal, QC, Canada). The explants were washed twice in complete culture medium (DMEM supplemented with 10% heat-inactivated FBS serum, 1% Pen-Strep, and 1% amphotericin) and maintained for 6 days under standard culture conditions (humidified atmosphere and 5% CO_2_). The complete culture medium was changed every three days.

Osteochondral explants were treated for 14 days in complete culture medium (control) or complete culture medium supplemented with LN (100 µg/mL). The treatment culture medium was changed every three days. The treatment was terminated by washing the explants in cold PBS, and the treated explants were fixed in Accustain (Millipore Sigma, Burlington, MA, USA) and decalcified in Osteosoft (Millipore Sigma, Burlington, MA, USA) before paraffin embedding.

### 2.6. Immunohistochemistry

The 5 µm sections of osteochondral explants and rabbit samples were rehydrated prior to immunohistochemistry by deparaffinization in xylene followed by sequential incubation in decreasing concentrations of alcohol and water. Sections were blocked using a VECTASTAIN ABC kit (Vector Laboratories, Burlingame, CA, USA), followed by overnight incubation with anti-POSTN antibody (Cat# 66491-1-IG, ThermoFisher, Waltham, MA, USA) (1:1000) at 4 °C in Tris–saline buffer containing 1% BSA. The secondary antibody and subsequent slide processing were performed using a VECTASTAIN ABC kit following the manufacturer’s guidelines. The substrate and development were processed with a 3,3′-diaminobenzidine peroxidase substrate kit (Vector Laboratories, Burlingame, CA, USA). Sections were counterstained with hematoxylin, dehydrated in sequential alcohol concentrations and Histo-Clear (ThermoFisher Scientific, Waltham, MA, USA), and mounted in Permount (ThermoFisher Scientific, Waltham, MA, USA). Positive cells were validated based on immunohistochemistry reactions.

### 2.7. Chondrocyte Isolation

Human normal and OA chondrocytes were recovered from knee cartilage (*n* = 3 and 4 donors, respectively) by sequential digestion with 0.125% Pronase (Wisent Bioproducts, Montreal, QC, Canada) for 30 min. After isolation, the cells were cultured in pellets or monolayers and expanded in complete culture media.

### 2.8. Chondrocyte Activation and Gene Expression

To determine the effects of LN on POSTN-induced gene expression, freshly isolated human normal and OA chondrocytes, cultured as micro-pellets at a density of 5 × 10^5^ cells/pellet, were treated for 6 days in 0.5 mL of DMEM supplemented with POSTN (20 μg/mL) in the presence or absence of LN (1.0 µg/mL or 100 µg/mL). Pellets treated with 1 µg/mL of SC or without LN for the same amount of time were used as controls. The total RNA was extracted using a total RNA mini kit (Geneaid Biotech Ltd., New Taipei City, Taiwan) according to the manufacturer’s instructions. Complementary DNA was synthesized using a superscript Vilo cDNA synthesis kit (ThermoFisher Scientific, Waltham, MA, USA). The human DNA was quantified using quantitative real-time PCR (ABI 7500 fast light cycler), CYBR green master mix (ThermoFisher Scientific, Waltham, MA, USA), and specific primers (Table 1). The relative mRNA expression levels were normalized against GAPDH.

### 2.9. β-Catenin Signaling and POSTN Expression

Human OA chondrocytes (*n* = 3 donors) were transferred to 6-well plates and cultured in complete culture medium. At 90% confluency, cells were serum deprived overnight and incubated in DMEM containing POSTN (20 μg/mL) and LN (0, 1.0, or 100 μg/mL) for 60 min at 37 °C. Control cells were incubated in DMEM alone for 10 min. Cells were lysed in RIPA (radio immunoprecipitation assay) buffer and protease cocktail II (Sigma-Aldrich, St. Louis, MO, USA) and phosphatase (ThermoFisher Scientific, Waltham, MA, USA) inhibitors. The lysate was electrophoresed on 4–20% gradient gels (Bio-Rad, Hercules, CA, USA) under reducing conditions and transferred to 0.2 μm PVDF membranes. Blots were probed with anti-β-catenin antibody (Cat# 32572, Abcam, Cambridge, UK) and GAPDH (Sigma-Aldrich, St. Louis, MO, USA) for normalization.

### 2.10. Peptide Docking

The peptide docking of the LN to the POSTN (crystal structure: 5yjg) was determined using the CABS–dock web server (http://biocomp.chem.uw.edu.pl/CABSdock/, accessed on 10 September 2022). The best prediction generated by CABS–dock was added to PyMOL ver 2.5.4 (Schrodinger, LLC, New York, NY, USA) to create the model.

### 2.11. Immunoprecipitation and POSTN Dissociation

Biotinylated LN (50 μg) and biotinylated SC (50 μg) were allowed to bind to Avidin-labelled agarose beads and were washed thrice in TBS and then incubated with 1 μg of POSTN for 1 h at room temperature. Beads were washed thrice in TBS and then boiled in Laemmli buffer containing DTT (Bio-Rad, Hercules, CA, USA). Western blotting was performed to identify the POSTN–LN interaction. Original figures can be found in Supplementary Materials. 

The dissociation of the POSTN was performed by incubating POSTN (0.5 μg) and LN or SC (1 μg) together for 1 h at room temperature. Samples were then electrophoresed on 4–20% gradient gels (Bio-Rad, Hercules, CA, USA) under nondenaturing conditions. Control samples included POSTN processed under nondenaturing and denaturing conditions (Laemmli buffer + 1 mM dithiothreitol + boiling at 100 °C).

### 2.12. Statistical Analysis

Data were analyzed using ANOVA followed by a post hoc Dunnett’s test for continuous variables or two-way ANOVA followed by a post hoc Dunnett’s multiple comparison test or a chi-squared test for dichotomous variables. A *p*-value of less than 0.05 was considered as statistically significant.

## 3. Results

### 3.1. LN Suppresses the Expression of POSTN in Human OA Cartilage

**Figure 1:**
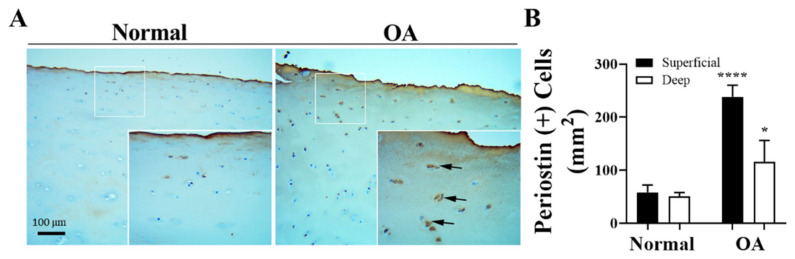
Increased expression of Postn in human OA cartilage. (**A**) Immunohistochemical analysis of human OA knee cartilage identified increased cell-associated POSTN in lesional areas when compared to normal cartilage (back arrows). OA knee cartilage, obtained from four donors ranging from 35 to 70 years of age and undergoing total knee arthroplasty, was sectioned and stained for POSTN with Vectastain reagents (Vector Laboratories, Burlingame, CA, USA). (**B**) Quantitation of periostin positively expressing chondrocytes in superficial and deep cartilage zones. Two-way ANOVA; comparison between normal and OA cartilages; **** *p* < 0.0001; * *p* < 0.05; *n* = 4.

**Figure 2:**
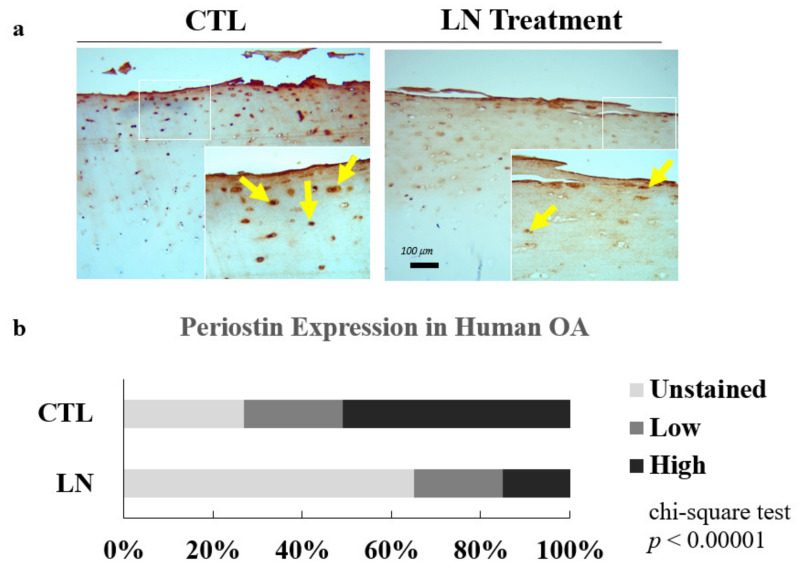
LN suppresses the upregulation of POSTN in human OA osteochondral explants: (**a**) Human OA osteochondral explants were treated with LN (100 µg/mL) or PBS (CTL) for 14 days. Changes in expression were determined by POSTN staining (arrows); (**b**) immunohistochemical analysis of POSTN expression in human OA samples, where the stains were divided into three categories: cells unstained with POSTN, cells with minimal Postn staining, and cells with saturated POSTN staining. Means ± SDs; *n* = 4 donors; chi-squared test.

Because *POSTN* is a gene of interest in cartilage repair, we investigated its expression in normal and OA cartilage sections by immunohistochemical staining [30,31]. Our results indicate increased expressions of POSTN in human OA cartilage sections when compared to normal cartilage (Figure 1A). Pericellular and cell-associated stainings of POSTN were observed mostly in superficial zones and, to some extent, the deep zone of the lesional OA cartilage. We observed significant increases in the detection of periostin positively expressing chondrocytes in OA cartilage (Figure 1B). In contrast, mainly the superficial zone exhibited staining in non-lesional areas of the same specimen (Figure 1 and Figure 2).

**Figure 3:**
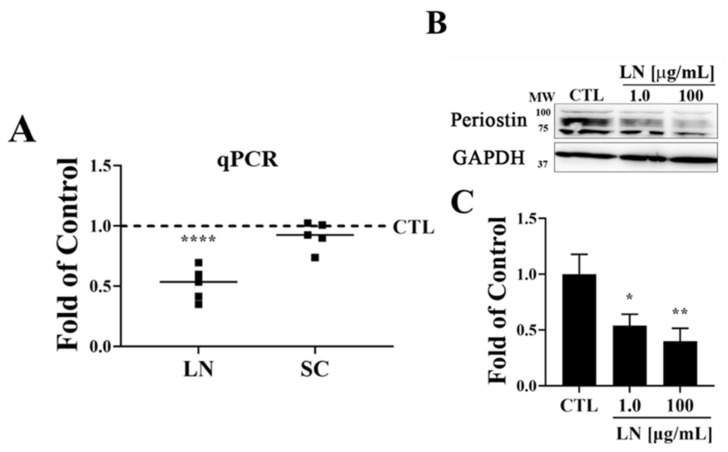
Effect of LN on POSTN expression in primary human osteoarthritis chondrocytes. (**A**) Freshly isolated human osteoarthritis (OA) chondrocytes were cultured as micro-pellets and treated for 6 days with LN at 1 µg/mL and 100 µg/mL. Quantitative real-time PCR was used to measure *POSTN* mRNA expression levels, normalized against GAPDH. (**B**) Representative western blot analysis showing the effects of the LN treatments (at 1 µg/mL and 100 µg/mL) on POSTN protein levels over a 3-day treatment period. GAPDH was used as the loading control. (**C**) Densitometric analysis of POSTN protein levels from western blots (panel B), normalized to GAPDH. Statistical significance is indicated as * *p* < 0.05; ** *p* < 0.01; and **** *p* < 0.0001 compared to the untreated control group.

To determine if LN regulates the expression of *POSTN* in human OA cartilage, we cultured human chondrocytes as micro-pellets in media supplemented with various concentrations of LN. Gene expression data indicate that *POSTN* is significantly downregulated in chondrocytes following LN treatment when compared to the control (Figure 3A). Furthermore, the immunoblot analysis of the LN-exposed human chondrocytes revealed that LN inhibits POSTN synthesis in chondrocytes (Figure 3B,C).

To determine whether LN can inhibit the expression of POSTN, we treated human OA osteochondral explants with LN for 14 days. The immunohistochemical analysis of the OA-treated cartilage sections indicated that LN inhibits POSTN expression in human OA cartilage (Figure 2). In Figure 2a, the immunohistochemical staining of the OA cartilage shows a decrease in POSTN expression following the LN treatment. Although it is possible that LN binding to Postn could result in epitope masking, the reduction in POSTN protein levels was further confirmed through western blot analysis (Figure 3). These analyses showed significant decreases in POSTN protein levels in LN-treated samples, indicating that the reduction observed in the IHC staining is because of the actual downregulation of the POSTN expression rather than epitope masking. Additionally, quantitative PCR (qPCR) data (Figure 3A) demonstrated a marked decrease in *POSTN* mRNA expression in response to LN treatment, further supporting the conclusion that LN reduces *POSTN* expression at the transcriptional level.

### 3.2. POSTN Suppression in an Experimental Model of OA by LN

**Figure 4:**
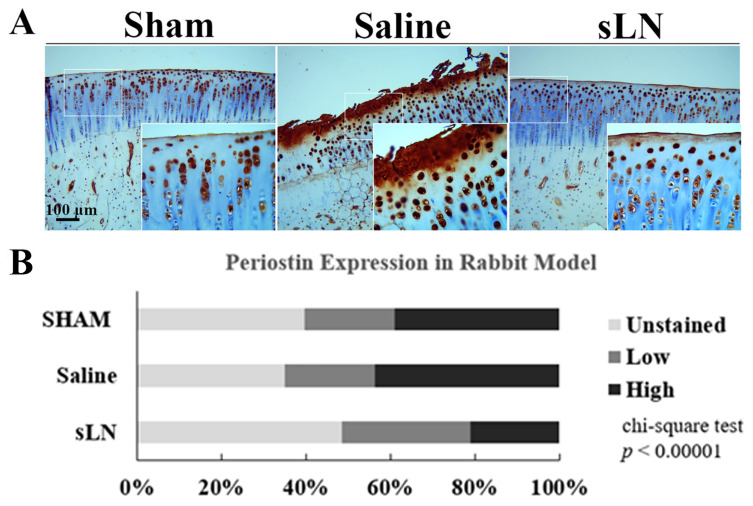
LN suppresses the upregulation of POSTN in a rabbit model of OA. Skeletally mature New Zealand white rabbits underwent unilateral anterior cruciate ligament transection (ACLT) of their left femorotibial joints to induce joint degeneration typical of OA. Beginning at 3 weeks postoperatively, and every three weeks thereafter for 12 weeks, either saline (1 mL) or sLN (100 µg in 1 mL of saline) was injected intraarticularly into the operated knee. Six additional rabbits underwent sham surgery but without ACLT or postoperative injections: (**A**) POSTN expression, as determined using immunostaining at 12 weeks, was significantly higher in the ACLT rabbits’ knee cartilage when compared with sham knees; (**B**) the immunohistochemical analysis of the POSTN expression in the rabbit model of OA, where the stains were divided into three categories: cells unstained with POSTN, cells with minimal POSTN staining, and cells with saturated POSTN staining. Statistical significance was assessed using a chi-squared test (*p* < 0.00001).

Furthermore, we conducted experiments to determine whether the expression of the POSTN is also increased in the cartilage of rabbits subjected to ACLT when compared with those undergoing a sham procedure. The ACL-operated knees treated with saline showed significantly higher POSTN protein expressions compared to sham-operated knees or those administered with LN (Figure 4). Interestingly, the LN-treated animals demonstrated decreased expressions of POSTN when compared to the sham group.

### 3.3. LN Decreases POSTN Signaling in Human OA Chondrocytes

**Figure 5:**
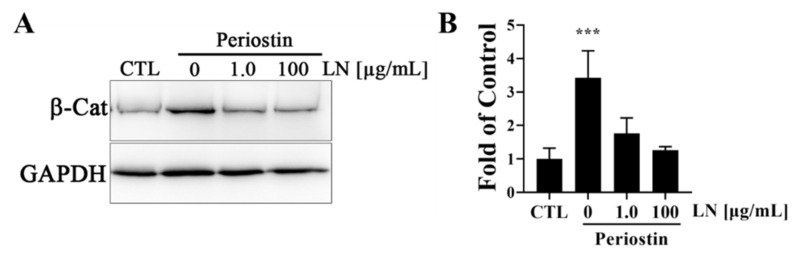
LN decreases POSTN signaling in human OA chondrocytes: (**A**) western blots demonstrating the inhibition of periostin-induced increases in β-catenin (β-Cat) accumulation by LN; (**B**) densitometry of blots presented in (**A**). ANOVA; post hoc Dunnett’s test; *** *p* < 0.0001; *n* = 3.

**Figure 6:**
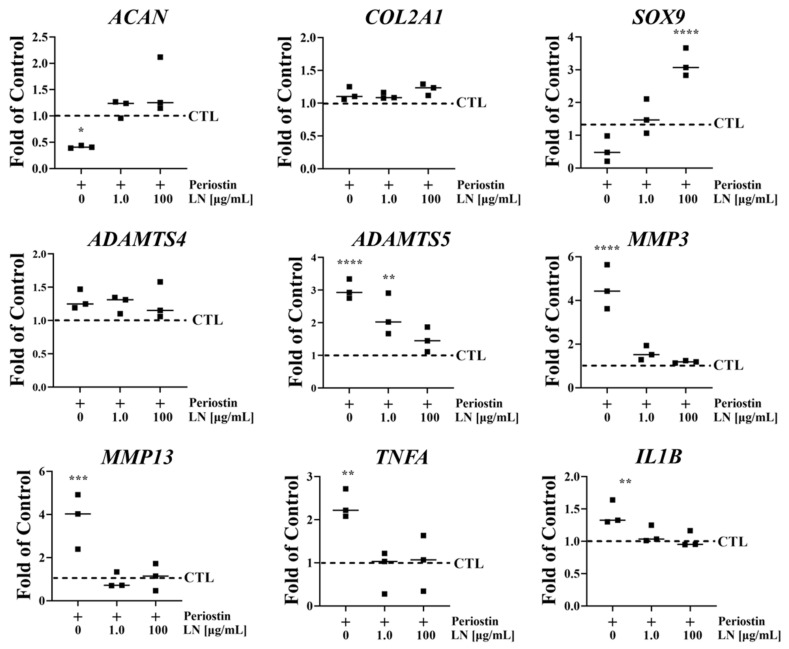
LN regulates periostin-induced gene expression in human OA chondrocytes. Chondrocyte pellets were treated with Link N (at 1 or 100 µg/mL) or PBS (CTL) for 6 days in the absence or presence of periostin (20 μg/mL). Gene expression was measured using qPCR. Means ± SDs; *n* = 3 donors; ANOVA; post hoc Dunnett’s multiple comparison test; **** *p* < 0.0001; *** *p* < 0.001; ** *p* < 0.01; * *p* < 0.05 in comparison with the control.

To determine whether POSTN could activate the Wnt/β-catenin pathway, which is known to be implicated in OA, human OA chondrocytes were incubated with POSTN for 60 min with or without LN and measured for the intracellular accumulation of β-catenin. As shown in Figure 5, in control chondrocytes where no POSTN was added, a faint but noticeable band was apparent in the immunoblots. There were significant increases in the accumulation of β-catenin in chondrocytes following incubation with POSTN when compared to controls (Figure 5A,B). The accumulation of β-catenin was dose dependently suppressed when POSTN was co-incubated with LN (Figure 5A,B). Additionally, LN was shown to decrease *POSTN*-induced gene expressions (Figure 6).

### 3.4. LN Interacts with POSTN and Induces Dissociation

**Figure 7:**
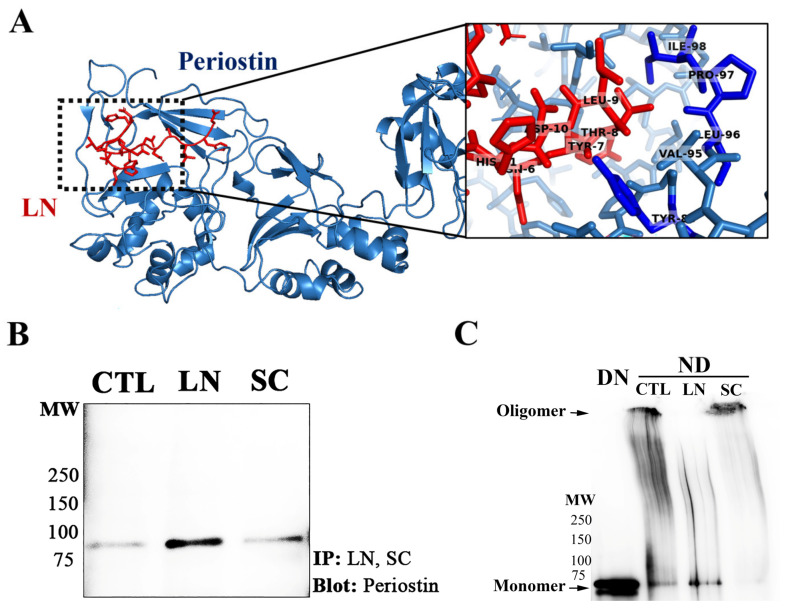
LN interacts with POSTN and induces dissociation: (**A**) The peptide docking of the LN to POSTN (crystal structure: 5yjg) was determined using the CABS–dock web server. The model was created using PyMOL (Schrodinger, LLC). POSTN residues known to be important in its dimerization are shown to interact with LN. (**B**) The immunoprecipitation (IP) of the LN with POSTN. Biotinylated LN or biotinylated scrambled LN (SC) was attached to Avidin-labelled agarose beads and then incubated with POSTN. Western blotting was performed to identify POSTN–LN interactions. Lane 1: CTL (PBS) with POSTN; lane 2: IP of LN with POSTN; lane 3: SC with POSTN. (**C**) POSTN incubated with LN dissociates dimer/oligomer formation.

Using in silico docking, LN was predicted to predominantly dock to the EMI domain of the POSTN (Figure 7A), interacting with several residues implicated in periostin dimerization. For instance, dimerization residues ILE98, PRO97, LEU96, and TYR83 (blue) are found to pair with residue THR8 of LN (red). Given the location and docking of the LN to periostin, it is possible that this may interfere with periostin dimerization/oligomerization. To determine if LN can physically interact with POSTN, we performed pull-down experiments.

Our results show that LN was capable for immunoprecipitating POSTN; however, when similar procedures were performed with SC, no enrichment was observed as compared to controls (Figure 7B). Additionally, under nondenaturing conditions, POSTN has been shown to exist as dimers/oligomers. When purified POSTN was incubated with LN and processed under nondenaturing conditions, the Postn primarily migrated at approximately 90 kDa (Figure 7C). The POSTN remained mostly oligomeric when incubated with the SC peptide.

## 4. Discussion

Elevated levels of POSTN in the cartilage are associated with OA. The present data indicate that LN suppresses the overexpression of *POSTN* in human OA cartilage and in an experimental animal model of OA. Furthermore, LN appears to interact with POSTN and induces dissociation, leading to a decrease in POSTN signaling in human OA chondrocytes via the activation of the Wnt/β-catenin pathway.

The findings of this study are consistent with previous research demonstrating the potential therapeutic effects of LN in cartilage repair and OA management. Prior studies have shown that LN can inhibit catabolic factors and stimulate matrix protein synthesis in cartilage [15,17,18,19,20,21,22,23]. In line with these reports, our data indicate that LN suppresses the overexpression of POSTN in OA cartilage and decreases POSTN-induced signaling in chondrocytes. Additionally, the ability of LN to modulate inflammation and reduce the catabolic effects of POSTN align with earlier studies [24,25]. However, our study expands on this understanding by specifically illustrating the interaction of LN with POSTN at a molecular level, revealing new insights into its role in mitigating OA progression.

The observed decrease in POSTN expression in Figure 2a could potentially be attributed to epitope masking caused by LN binding to POSTN. However, we addressed this concern by conducting western blot and qPCR analyses, which both indicated a significant reduction in POSTN protein and mRNA levels in LN-treated samples (Figure 3A,B). These findings support the interpretation that LN exerts its effects by downregulating POSTN expression rather than merely masking the epitope. Thus, these results suggest that LN actively inhibits POSTN synthesis at both the protein and mRNA levels, underscoring its potential therapeutic impact on mitigating OA progression.

POSTN has been shown to have catabolic activity in chondrocytes. Although the exact mechanism is not fully understood yet, existing research has unveiled some aspects of the impact of POSTN on chondrocyte function [32,33,34,35,36]. A study has shown that POSTN induces inflammatory cytokines and MMPs via the activation of various signaling pathways, including PI3K/Akt, Wnt/β-catenin, and nuclear factor kappa-light-chain-enhancer of activated B cells (NF-κB) [32]. Its expression increases following joint injury, resulting in cartilage degeneration, and it can also promote cartilage degeneration in patients with OA [32]. Other studies have shown that POSTN expression is induced by TGF-β in osteoblasts and fibroblasts [33,34] and that it can signal through αvβ3/αvβ5 integrins in various cell types [33,34,35,36]. Interestingly, as the nature of the POSTN receptor(s) in chondrocytes remains unexplored, a group of researchers has demonstrated POSTN interacting with discoidin domain receptor 1 (DDR1), a type I transmembrane protein that is composed of a cytoplasmic C-terminal tyrosine kinase domain and is expressed in human OA chondrocytes. Further research was able to show that the interaction between DDR1 and POSTN can activate inflammatory-signaling pathways. Therefore, Han et al. explored the involvement of the POSTN and DDR1 interaction in OA cartilage [14]. They found that POSTN-induced AKT and β-catenin signaling were blocked by inhibiting DDR1 kinase, and lower levels of MMP-13 expression were observed. These findings suggested that POSTN induces a catabolic effect on OA by interacting with DDR1, activating PI3K-AKT- and Wnt/β-catenin-signaling pathways. This interaction results in an increase in AKT phosphorylation and β-catenin stabilization, as well as the upregulation of MMPs and proinflammatory cytokines, such as MMP 1, 3, and 13 and IL-1β [14].

In other studies, POSTN was reported to be overexpressed in lesions of both human OA and different rodent models of OA. Notably, its presence has been linked to the induction of MMP-13 and ADAMTS-4 (a disintegrin and metalloproteinase with thrombospondin motifs-4) expressions [37,38,39,40,41], two enzymes known to play important roles in the degradation of the cartilage matrix. Additionally, POSTN upregulation in cartilage from different OA patients showed considerable variation, which could be because of the heterogeneity of the disease. Interestingly, experiments utilizing the siRNA-mediated knockdown of endogenous POSTN blocks have revealed a consequential effect on constitutive MMP-13 expression, which is consistently corroborated with the activation of the Wnt/b-catenin-signaling pathway. These results have implications in relation to cartilage repair and point to POSTN being involved in exacerbating cartilage degeneration in OA by upregulating MMP-13 through canonical Wnt signaling [4,30,31].

In a study by Tajka et al. [42], POSTN levels in the synovial fluid increased with OA severity. Additionally, increases in POSTN expression have been found in human OA cartilage from knee joints and in a murine model of OA following destabilization of the medial meniscus [32,37]. Several studies have shown that mice with POSTN deficiency developed less-severe OA compared to controls [43]. Similar results were observed in age-related mice models [43] These results suggest that the lost of function of POSTN POSTN can protect mice against posttraumatic and age-related OA and could be a therapeutic target to delay or prevent OA.

The ability of POSTN to promote inflammation by activating NF-kB signaling has been reported previously in keratinocytes [44,45,46]. Other studies showed that NF-kB induces the expression of POSTN in kidney cells to promote renal injury [47]. Collectively, these studies suggest a strong link between POSTN and NF-kB. Our recent findings showed that LN can suppress NF-kB, providing a strong rationale to investigate POSTN as a potential therapeutic target for the treatment of OA.

In healthy cartilage, POSTN expression is low; however, in OA or induced OA, as demonstrated in in vivo models, POSTN expression is upregulated. Suppressing the function of POSTN may regulate cartilage degeneration and protect against OA. LN has been shown to regulate cartilage degeneration by enhancing matrix protein synthesis and inhibiting catabolic enzyme and IL-1β functions. The results from this study indicate a role for LN in regulating POSTN in cartilage as well. Future work is needed to determine the optimal window and delivery mechanism of LN in regulating POSTN in the treatment of OA.

POSTN is known to exist in multiple splicing variants, particularly in the C-terminal region, which can result in structural differences that may affect its biological function and interaction with therapeutic agents [6,48,49]. Although this study focused on a specific variant of POSTN that is commonly upregulated in OA cartilage, the abilities of LN to bind and inhibit other splicing variants have not been fully explored. Given the conservation of the LN binding site in the dimerization region [6], it is possible that LN could interact with multiple POSTN variants. However, the structural differences in other splicing variants could influence the binding affinity and inhibitory effects of LN [48,49]. Further studies are needed to evaluate the efficacy of LN across different POSTN variants, particularly those with alternative C-terminal structures.

The similarity in POSTN sequences, between rabbits and humans, at the LN binding site underscores the relevance of the rabbit OA model for studying the therapeutic potential of LN. The positive outcomes observed in the rabbit model, including the reduction in POSTN expression and cartilage protection, are promising indicators of the applicability of LN in human clinical settings. Nonetheless, further studies, including clinical trials, are essential to confirm the translatability of these findings to human patients with OA.

Although our study demonstrated the therapeutic efficacy of LN in reducing POSTN expression and protecting cartilage in an OA model, the specific distribution and penetration depth of the LN within the cartilage tissue following intraarticular administration were not directly measured. Previous studies on similar peptides have suggested that LN can penetrate compromised cartilage effectively, as indicated by the observed biological effects [17,18,19,28].

LN specifically targets extracellular POSTN, which is highly involved in the pathological processes of OA. This targeted action distinguishes LN from other therapeutic strategies, like siRNA or antisense oligonucleotides, which must enter cells to suppress periostin expression at the mRNA level. The extracellular action of the LN offers several advantages, including reduced complexity, a potentially better safety profile because of fewer off-target effects, and more immediate therapeutic effects. Additionally, the extracellular mechanism of action LN complements intracellular approaches, offering the possibility of combination therapies that target POSTN at multiple levels.

Our study demonstrated the effective suppression of POSTN in OA-affected tissues by LN without observable adverse effects in our animal models. Given that periostin is also expressed at physiological levels in healthy tissues, it is critical to consider the potential side effects of its suppression. However, our data suggest that the inhibitory effects of LN are predominantly observed in the context of pathological overexpression in OA. This selective suppression indicates that LN may not significantly impact the baseline physiological functions of POSTN.

## 5. Conclusions

We provide evidence for the direct role of LN in inhibiting POSTN expression and signaling in OA. These results suggest that the suppression of POSTN by LN may provide a therapeutic target in the treatment of OA.

## Figures and Tables

**Table 1:** List of Primers for PCR.

Genes	Primer Sequence
*POSTN*	**F:** 5′-TCTGTTTTAGACCCTTTTTCATTGTCCTTCT-3’ **R:** 5′-CTGCCATTTATGCTTAATTCCTTATTCTTGTG-3’
*ACAN*	**F:** 5′-TGAGTCCTCAAGCCTCCTGT-3’ **R:** 5′-CCTCTGTCTCCTTGCAGGTC-3’
*COL2A1*	**F:** 5′-ATGACAATCTGGCTCCCAAC-3’ **R:** 5′-CTTCAGGGCAGTGTACGTGA-3’
*SOX9*	**F:** 5′-TTCATGAAGATGACCGACGA-3’ **R:** 5′-CGCTCTCCTTCTTCAGATCG-3’
*ADAMTS4*	**F:** 5′-TCCTGCAACACTGAGGACT-3’ **R:** 5′-GGTGAGTTTGCACTGGTCC-3’
*ADAMTS5*	**F:** 5′-ACAAGGACAAGAGCCTGGAA-3’ **R:** 5′-ATCGTCTTCAATCACAGCACA-3’
*MMP3*	**F:** 5′-GGCAGTTTGCTCAGCCTATC-3’ **R:** 5′-GAGTGTCGGAGTCCAGCTT-3’
*MMP13*	**F:** 5′-TAAGGAGCATGGCGACTTC-3’ **R:** 5′-GGTCCTTGGAGTGGTCAAG-3’
*TNFA*	**F:** 5′-ACCACGCTCTTCTGCCT-3’ **R:** 5′-TACAACATGGGCTACAGGCTT-3’
*GAPDH*	**F:** 5′-GCTCTCCAGAACATCATCCCTGCC-3’ **R:** 5′-CGTTGTCATACCAGGAAATGAGCTT-3’

## Data Availability

Data is contained within the article or Supplementary Materials.

## Supplementary Materials

The following supporting information can be downloaded at: https://www.mdpi.com/article/10.3390/biom14111469/s1, Figure S1. Effect of LN on Postn expression in primary human osteoarthritis chondrocytes. Representative Western blot showing the effect of LN treatment (1 μg/mL and 100 μg/mL) on Postn protein levels over a 3-day treatment period. Black arrow indicates periostin expression. GAPDH was used as the loading control. Original images of Figure 3. Figure S2. LN decreases Postn signaling in human OA chondrocytes: Western blots demonstrating inhibition of Periostin-induced increases in β-catenin (β-Cat) accumulation by LN. Original images of Figure 5. Figure S3. LN interacts with Postn and induces dissociation: Immuno-precipitation (IP) of LN with Postn. Biotinylated LN or biotinylated scrambled LN (SC) was attached to Avidin-labelled agarose beads and then incubated with Postn. Western blotting was performed to identify Postn-LN interaction. Lane 1: CTL (PBS) with Postn; lane 2: IP of LN with Postn; lane 3: SC with Postn. Original image of Figure 7.
