# Supplementary material for: Advances in the Regulation of Periostin for Osteoarthritic Cartilage Repair Applications
Source: Biomolecules. 2024 Nov 18;14(11):1469. doi: 10.3390/biom14111469 (PMC11592007; doi:10.3390/biom14111469)
Supplement: Supplementary file 1 [file biomolecules-14-01469-s001.zip › biomolecules-3183514-supplementary.pdf]

Supplemental Material – Western Blots

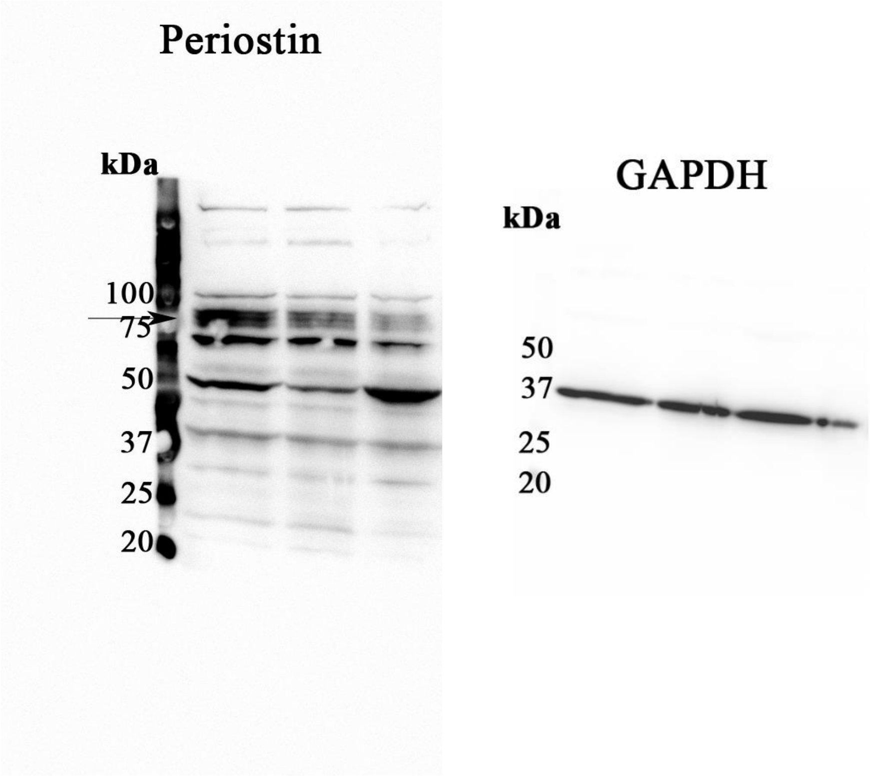

Figure 3. Effect of LN on Postn expression in primary human osteoarthritis chondrocytes.

Representative Western blot showing the effect of LN treatment (1 µg/mL and 100 µg/mL) on Postn protein levels over a 3-day treatment period. Black arrow indicates periostin expression. GAPDH was used as the loading control.

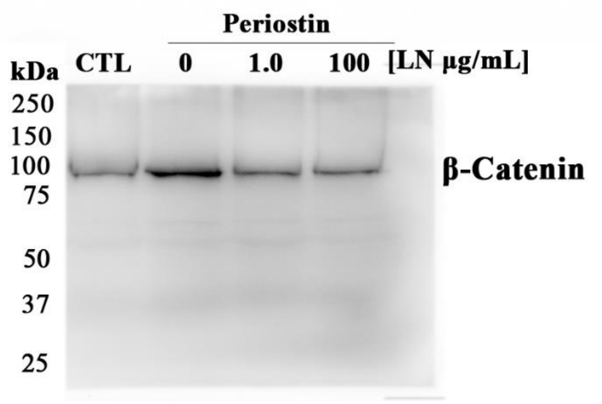

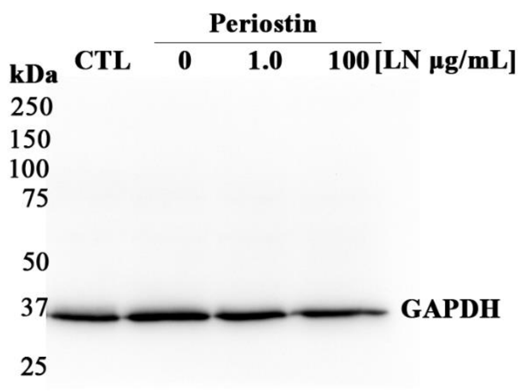

Figure 5. LN decreases Postn signaling in human OA chondrocytes: Western blots demonstrating inhibition of Periostin-induced increases in β-catenin (β-Cat) accumulation by LN.

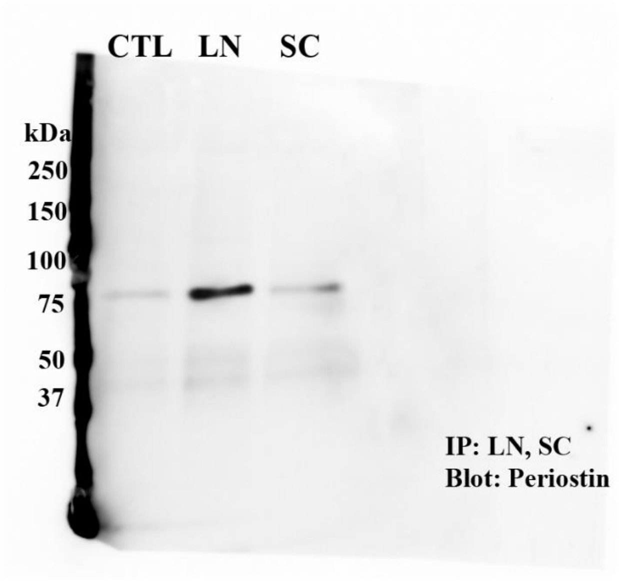

Figure 7. LN interacts with Postn and induces dissociation: Immuno-precipitation (IP) of LN with Postn. Biotinylated LN or biotinylated scrambled LN (SC) was attached to Avidin-labelled agarose beads and then incubated with Postn. Western blotting was performed to identify Postn-LN interaction. Lane 1: CTL (PBS) with Postn; lane 2: IP of LN with Postn; lane 3: SC with Postn.
